# Diagnosis and treatment of solid pseudopapillary neoplasm of the pancreas in children: A report of 18 cases

**DOI:** 10.3389/fped.2022.899965

**Published:** 2022-08-08

**Authors:** Ayiguzaili Maimaijiang, Haiyun Wang, Wanfu Li, Yaqi Wang

**Affiliations:** Department of Pediatric Surgery, First Affiliated Hospital of Xinjiang Medical University, Urumqi, China

**Keywords:** diagnosis, treatment, solid pseudopapillary neoplasm, children, surgical approach

## Abstract

**Purpose:**

To explore the incidence, imaging and treatment of solid pseudopapillary tumor of pancreas in children, and Summarize the experience of treatment.

**Methods:**

The clinical data of 18 children with a solid pseudopapillary tumor of the pancreas treated in our hospital from January 2012 to June 2021 were analyzed retrospectively. The age range was 8–16 years old, the average age was 11.67 years old, and the median age was 11.5 years old, namely, three boys with an average age of 10 years old and 15 girls with an average age of 12 years old. In total, two cases were admitted to the hospital because of trauma, seven cases were found in physical examination, and nine cases were admitted with the abdominal pain as the main complaint. Enhanced CT examination was performed before operation, nuclear magnetic resonance examination and abdominal ultrasound examination were performed in some children, and plain CT scan was performed after operation.

**Results:**

Solid pseudopapillary tumor of the pancreas can occur in all parts of the pancreas, especially in the body and tail of the pancreas. Among the 18 cases, SPN occurred in the head of pancreas in 5 cases (27.78%), the neck of pancreas in 2 cases (11.11%), and the body and tail of pancreas in 11 cases (61.11%). All the 18 children were treated by operation. among them, 4 cases underwent choledochal pancreatico duodenectomy (1 case), 4 cases underwent pancreaticoduodenectomy combined with splenectomy (3 cases), 6 cases underwent spleen-preserving pancreatectomy / tail pancreatectomy (1 case), 3 cases underwent enucleation of pancreatic tumor due to exogenous growth, 1 case underwent laparoscopic partial pancreatectomy and pancreaticoenterostomy. Laparotomy was performed in 12 cases and endoscopic surgery in 6 cases. Postoperative pathology confirmed solid pseudopapillary tumor of the pancreas. None of the patients received radiotherapy and chemotherapy after operation and were followed up for 6 months to 2 years. There was no recurrence, metastasis or pancreatic dysfunction.

**Conclusion:**

Solid pseudopapillary tumor of pancreas in children is a rare, low-grade malignant solid tumor with no specific clinical manifestations and laboratory examinations. Preoperative diagnosis mainly depends on enhanced CT. Surgical resection of tumor is a reliable treatment, and the specific operation is mainly based on the experience of the chief surgeon, the location of the tumor and the invasion of surrounding tissue. At present, there is no evidence of the effectiveness of other treatment options, and surgical resection of the tumor has a good prognosis.

## Introduction

Solid pseudopapillary neoplasm (SPN) of the pancreas is a rare pancreatic tumor with malignant potential, accounting for ~1%−3% of the pancreatic tumors (1, 2). The disease was first described by Frantz in 1959 and was successively named Frantz tumor, papillary solid tumor, papillary cystic tumor, poorly differentiated papilloma, and papillary epithelioma. The WHO officially named it a solid pseudopapillary neoplasm of the pancreas in 1996, or PST for short (3). In 2010, the WHO updated the disease-as-solid pseudopapillary neoplasm of the pancreas abbreviated as SPN (4). The disease often occurs in women of childbearing age, and it is rare in children, but with the progress of medical imaging, the detection rate of SPN in children has increased (5). SPN has no specific clinical manifestations, so it is difficult to diagnose before surgery (6). Although the surgical treatment of SPN has a high cure rate and a good prognosis, active surgery is recommended at home and abroad. This study analyzed the diagnosis and treatment process of 18 children with SPN who received surgical treatment, summarized the diagnosis and treatment experience, and reported the following.

## Materials and methods

### Study design

This was a single-center retrospective case series. All the surgeries were carried out by members of the same surgical team. Medical records were analyzed by two surgeons (AY and WH) for data, namely, radiological findings, operation records, and medical files. The clinical data of 18 patients with SPN in the First Affiliated Hospital of Xinjiang Medical University from January 2012 to June 2021 were analyzed retrospectively. The clinical manifestations, imaging features, tumor location, pathology, surgical treatment, and prognosis were analyzed to summarize the treatment experience. The main postoperative follow-up was an outpatient follow-up from 6 months to 2 years. Abdominal routine ultrasound or plain CT examination was performed at 6, 12, and 24 months after the operation. During the follow-up period, there were no long-term complications, such as tumor recurrence, metastasis, diabetes with dyspepsia, or pancreatic dysfunction. There was no significant difference in growth and development between children and children of the same age. Patients who were lost to follow-up or not willing to participate in the present study were also excluded. All the enrolled patients were informed and consented to about this study after permission from the Ethics Committee of our institute.

### Preoperative imaging examination

All the patients with SPN were examined by the pancreatic computed tomography (CT), magnetic resonance imaging (MRI), or ultrasound after admission. According to the imaging findings, the tumors were divided into three types: cystic, solid, and cystic-solid. At the same time, the relationship between the lesion and the pancreas (such as the head of the pancreas, the neck of the pancreas, the body of the pancreas, and the tail of the pancreas) and whether adjacent organs were involved were recorded. The tumor diameter was defined as the maximum cross-sectional diameter of the tumor on CT/MRI and measured.

### Surgical procedure

Statistics on the methods of laparotomy and laparoscopic surgery were made by consulting surgical records. All the 18 children were treated by operation, which depends on the location, size, and close relationship with the surrounding organs and tissues of the tumor. These include local resection of the focus, pancreaticoduodenectomy, resection of the head of the pancreas with preservation of the duodenum, resection of the body and tail of the pancreas, resection of the body and tail of the pancreas + splenectomy, surgical biopsy, lymph node dissection and pancreatic focus combined with other operations.

### Postoperative complications

The judgment of postoperative complications was based on the postoperative course records and the results of postoperative blood routine, biochemical routine, drainage fluid biochemistry, and bacterial culture. According to the Clavien-Dindo classification of the postoperative complications (7), postoperative pancreatic fistula (POPE), postoperative abdominal hemorrhage, abdominal infection, lymphatic leakage, etc. The diagnosis and classification of POPF were based on the reference standard of the International Study Group of Pancreatic Surgery (ISGPS) 2016 (8). Biochemical leakage was defined as an increase in amylase in drainage fluid 3 times or more than the normal value on the 3rd day after the operation (9). It is stipulated that the recording time of postoperative complications is up to the day before discharge or on the day of readmission.

### Statistical analysis

Statistical analysis was performed using SPSS 25.0 statistical software (IBM, USA). A descriptive study was used to study the clinical characteristics of the patients. The measurement data are expressed as the mean ± SD, and the counting data are expressed as a percentage (%).

## Results

### Clinical data

Of the 18 patients, three (16.7%) were males, and 15 (83.33%) were females, with an average age of 11.6 years (8–16 years). Among the 18 children, two (11.11%) were found after admission because of the trauma, and seven (38.89%) were found during physical examination. A total of nine (50.00%) cases were admitted to the hospital with abdominal pain as the main complaint. The nature of abdominal pain was intermittent epigastric pain and no radiation pain elsewhere, of which three cases were accompanied by vomiting. In the physical examination of admission, the abdominal mass was palpable in four (22.22%) cases, which was hard, had a poor range of motion, and had no tenderness (Table 1).

**Table 1:** Basic clinical characteristics of the patient.

**Clinical features**	**Frequency, Mean ±SD, Median, %**
**Gender**
Male	3 (16.7%)
Female	15 (83.33%)
Age, years	11.67 (8–11)
**Symptoms**
Without specific symptoms	9 (50.00%)
Abdominal pain	7 (38.89%)
Abdominal mass	4 (22.22%)
Posttrauma	2 (11.11%)
**Tumor location**
Head	5 (27.78%)
Neck	2 (11.11%)
Body and tail	4 (22.22%)
Tail	7 (38.89%)
Tumor size, cm	6.7 (3.2–11)
**Radiologic features**
Cystic	5 (27.78%)
Solid	7 (38.89%)
Mixed	6 (33.33%)

**Figure 1:**
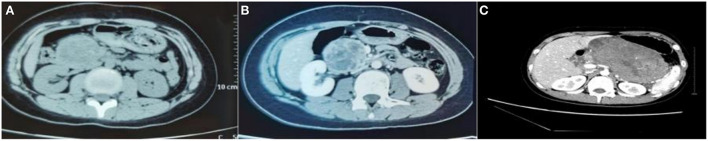
CT image of an exophytic SPN located in the head of the pancreas. **(A)** Plain CT scan of the abdomen showed a tumor in the head of the pancreas, showing an exophytic growth. **(B)** On contrast-enhanced CT, irregular separation can be seen in the cystic part of the cyst, and fibrous component enhancement can be seen in the cyst fluid, showing a “floating cloud sign”. **(C)** CT images of patients with SPN rupture caused by trauma.

**Figure 2:**
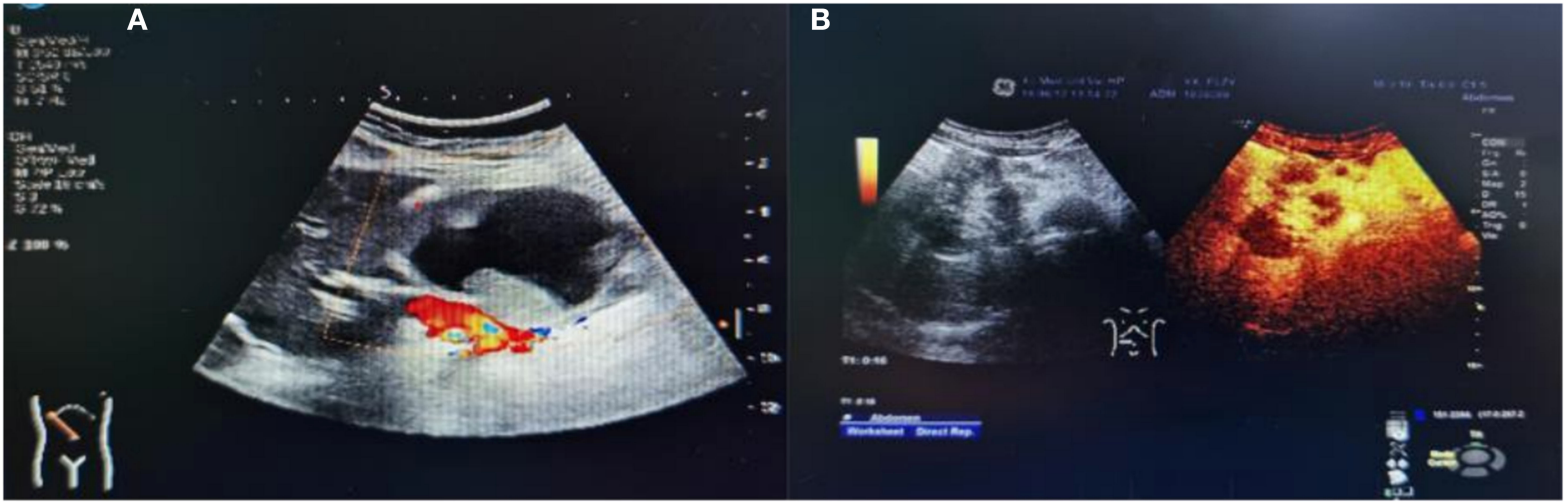
Ultrasound images of children with SPN. **(A)** Conventional ultrasound. **(B)** Contrast ultrasound.

All 18 children underwent enhanced CT examination before the operation, showing solid, cystic solid, or cystic round-like masses, namely, seven cases (38.89%) with solid or solid components, six cases (33.33%) with cystic solid components, and five cases (27.78%) with cystic or cystic components. The tumor was located in the head of the pancreas in five cases (27.78%), the neck of the pancreas in two cases (11.11%), and the body and tail of the pancreas in 11 cases (61.11%). A total of 11 cases of SPN were considered to be diagnosed after perfecting CT, and the sensitivity was 61.11%. The typical CT features were cystic-solid mass, low density, or isodensity on the solid part (Figure 1A). On contrast enhanced CT, there was mild-to-moderate enhancement, irregular separation was seen in the cystic part, and fiber component enhancement was seen in the cystic fluid, showing a “floating cloud sign” (Figure 1B). MRI examination was performed in five cases, and the diagnosis of SPN was considered in three cases, with a sensitivity of 60.0%. Among the five cases who underwent routine ultrasound examination, only one case considered the diagnosis of SPN, and the diagnostic sensitivity was 20.0% (Figure 2A). Among the seven cases combined with contrast-enhanced ultrasound, five cases were considered to be diagnosed as SPN, and the diagnostic sensitivity was 71.43% (Figure 2B). The overall sensitivity of preoperative imaging was 83.33%.

### Treatment regimen

**Figure 3:**
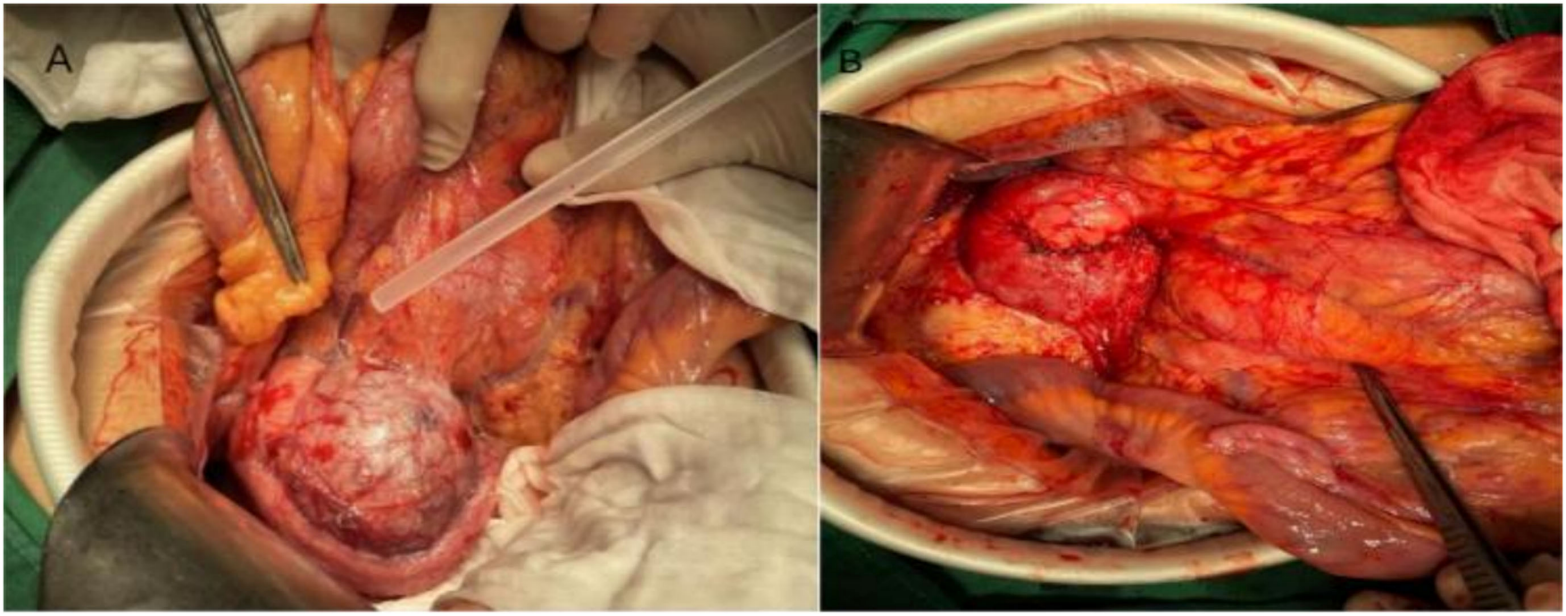
Intraoperative image of exophytic SPN in the head of pancreas. **(A)** Exogenous SPN in the head of the pancreas. **(B)** Picture after tumor enucleation.

Of the five children with tumors located in the head of the pancreas, four underwent choledochal pancreaticoduodenectomy (one case was performed under laparoscopy). Tumor enucleation was performed in one case because the tumor was exophytic with an intact capsule (Figures 3A,B). Among the 11 children with tumors located in the body and tail of the pancreas, three patients underwent laparoscopic pancreatectomy combined with splenectomy because the tumor surrounded the splenic vessels. In total, one case of SPN rupture was caused by trauma, and the ruptured tumor was found to have serious adhesion with surrounding tissues during operation. So pancreatectomy combined with splenectomy was performed (Figure 1C). Spleen-preserving resection of the body and tail of the pancreas was performed in six cases (laparoscopic operation in one case). Only one patient underwent enucleation of the pancreatic tumor because the tumor was exogenous and the capsule was intact. In the two cases of tumors located in the neck of the pancreas, one case underwent tumor removal because of tumor exogeny and an intact capsule, and 1 case underwent laparoscopic pancreatectomy with preservation of the duodenum. The specific tumor data and operation methods are shown in Table 2.

**Table 2:** Summary of basic information.

**Number**	**Gender**	**Location**	**Size (cm)**	**Boundary**	**Nature (cystic, solid)**	**Tumor marker**	**Mode of operation**
1	Female	Body and tail of pancreas	5.4 × 4.2 × 3.2	clear	Solid	Normal	Resection of body and tail of pancreas
2	Male	Body and tail of pancreas	6.3 × 5 × 3	clear	Cystic mainly	Normal	Resection of body and tail of pancreas + splenectomy
3	Female	Body and tail of pancreas	8.5 × 7.5 × 4.2	Enclose the splenic vessels	Solid mainly	Not checked	Laparoscopic resection of body and tail of pancreas plus splenectomy
4	Female	Body and tail of pancreas	5 × 5 × 2	clear	cystic	Normal	Laparoscopic resection of tail of pancreas
5	Female	Neck of pancreas	4 × 3.2 × 2.6	clear	Solid	Normal	Enucleation of pancreatic tumor
6	Female	Neck of pancreas	3.5 × 3.5 × 3	Clear	Solid mainly	Normal	Laparoscopic partial pancreatectomy and pancreaticoenterostomy
7	Female	Head of pancreas	5.7 × 5.3 × 5	clear	Cystic mainly	CA72-4 rises	Enucleation of pancreatic tumor
8	Female	Head of pancreas	7.5 × 6 × 4.5	clear	Cystic mainly	Normal	Pancreaticoduodenectomy with preservation of common bile duct
9	Female	Head of pancreas	8 × 6 × 5	Clear	Cystic mainly	Normal	Pancreaticoduodenectomy with preservation of common bile duct
10	Female	Head of pancreas	5.5 × 5 × 3.5	Invasion of the serous layer of the duodenum	Cystic mainly	Normal	Laparoscopic pancreaticoduodenectomy with preservation of common bile duct
11	Male	Head of pancreas	9 × 7 × 7	Clear	Solid	Normal	Pancreaticoduodenectomy with preservation of common bile duct
12	Female	Tail of pancreas	5.5 × 4 × 4	clear	Cystic mainly	Normal	Resection of tail of pancreas
13	Male	Tail of pancreas	4 × 3 × 2.5	Clear	Solid mainly	Not checked	Resection of body and tail of pancreas
14	Female	Tail of pancreas	11 × 11 × 6	Enclose the splenic vessels	Solid	Normal	Laparoscopic resection of body and tail of pancreas plus splenectomy
15	Female	Tail of pancreas	8.5 × 6.5 × 4.5	Enclose the splenic vessels	Solid	Normal	Laparoscopic resection of body and tail of pancreas plus splenectomy
16	Female	Tail of pancreas	3.2 × 3 × 2	Clear	Solid mainly	Not checked	Resection of tail of pancreas
17	Female	Tail of pancreas	10 × 10 × 8	Clear	cystic	Normal	Resection of body and tail of pancreas
18	Female	Tail of pancreas	10 × 8 × 5.5	Clear	Cystic & solid	Normal	Enucleation of pancreatic tumor

### Postoperative conditions

The postoperative hospital stay was 5–15 days, and complications occurred in two cases (11.11%). Among them, one case had pancreatic fistula, which was improved after fasting water, enzyme inhibition, and drainage. In total, one case had incomplete intestinal obstruction, which was improved after fasting water and acid inhibition treatment. No complications were found in other cases.

### Postoperative routine pathology

**Figure 4:**
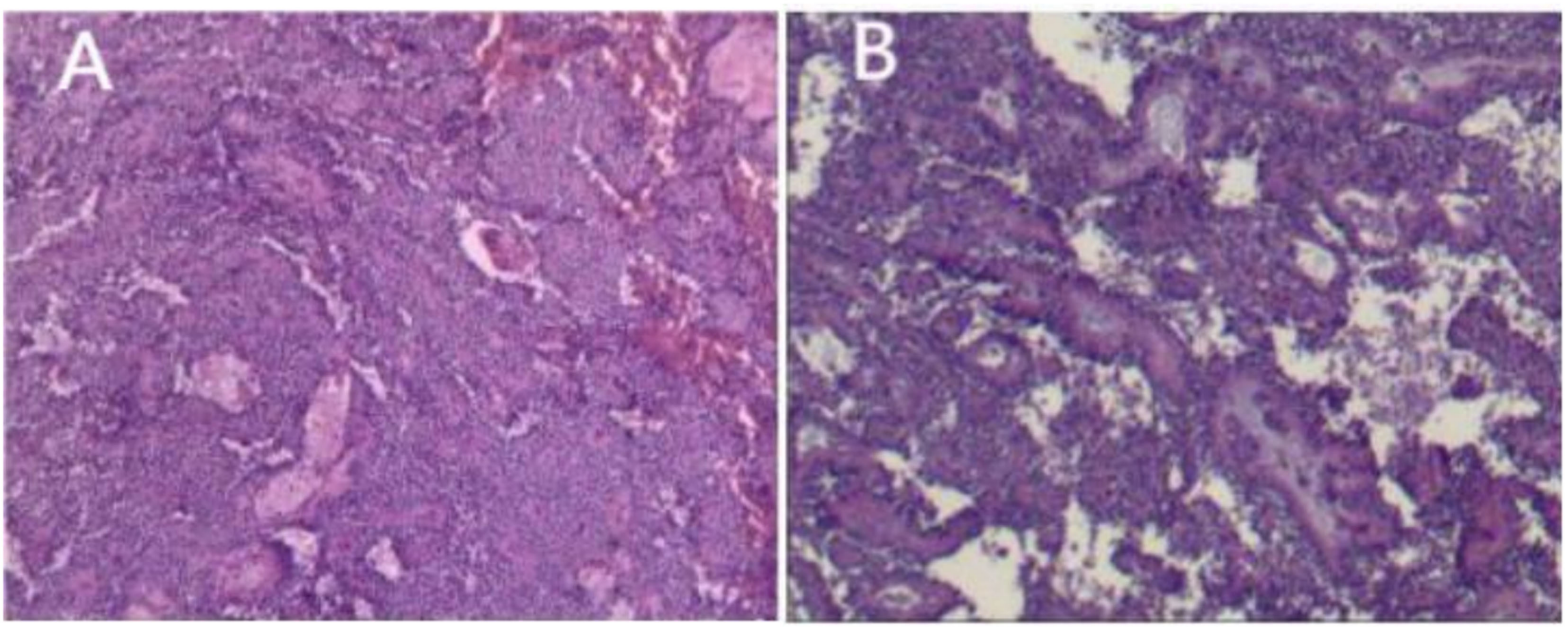
SPN routine pathology. **(A)** The pseudopapillary area of the tumor is formed by the tumor cells lining blood vessels, and hemorrhage can be seen. **(B)** Cystic and solid, and necrosis can be seen.

All the 18 children achieved complete resection of the tumor. The average maximum diameter of the tumor was 6.7 ± 2.43 cm. Ten cases (55.56%) had intact capsules, and four cases (22.22%) had peripheral tissue invasion and lymph node dissection, but no metastasis was detected. It is generally a round or quasi-round mass, the surface is smooth, the capsule is intact, and the section can be solid, cystic, or cystic (10, 11). The solid part is mostly gray, grayish white, or grayish red, and is crisp (12). If it is cystic and solid, it can be seen that the section is irregularly lobulated and separated, and the inner wall is rough; if the tumor is ruptured or bleeding, dark red bloody fluid can be seen in the capsule, and brown gravel-like substance can be seen in the capsule if old bleeding occurs (13) (Figures 4A,B).

## Discussion

Solid pseudopapillary neoplasm can occur in all parts of the pancreas, mostly, in the body and tail of the pancreas (14). Of the 18 cases in this group, 11 occurred in the body and tail of the pancreas, accounting for 61.11%. SPN usually occurs in women of childbearing age, and some studies have suggested that it is related to female hormones (15). In this group of cases, the male-to-female ratio was 1:5, which is in line with the general rule that the disease often occurs in women. SPN is potentially malignant, and its malignant features include vascular infiltration, nerve invasion, and deep invasion of the surrounding tissues (16). SPNs rarely have distant metastases, such as liver metastases, but there are also reports of ovarian and thyroid metastases (17–19). The incidence of the disease in childhood is less than that in adults, but with the development of medical imaging, the detection rate of the disease in children has increased. The disease usually has no clinical manifestations at the initial stage. With the growth of the tumor, symptoms or signs such as abdominal pain, nausea, vomiting, abdominal distension, and abdominal mass caused by the compression or invasion of surrounding organs gradually appear, but there is no specificity. With the growth of the tumor, symptoms or signs such as abdominal pain, nausea, vomiting, abdominal distension, and abdominal mass caused by compression or invasion of surrounding organs gradually appear.

Solid pseudopapillary neoplasm has no specific tumor markers, and other routine laboratory tests, such as blood glucose and amylase, are not special, which have no diagnostic significance for the disease. At present, the preoperative diagnosis of the disease mainly depends on CT and MRI. Its special pathological manifestations are solid, cystic-solid, and cystic structures, and also its lack of blood supply (20). It is shown as a mixed density shadow in CT, with delayed enhancement in enhancement, and the typical manifestation is the “floating cloud sign” (21). CT scan intuitively judges the relationship between the tumor and surrounding tissue, peripheral lymph node enlargement, and distant metastasis. Enhanced CT or CTA has a unique value in judging vascular invasion. MRI has higher tissue resolution than CT and has more advantages in judging the relationship between tumor tissue and the bile duct and pancreatic duct (22). It is of high value in the operation design and diagnosis of SPNs with complex components. The ultrasonic examination is subjective, and the diagnostic level is greatly affected by the examination of physician's personal technology, experience, and equipment, so the diagnostic sensitivity is not high. With the development of ultrasound technology and because of its advantages, such as noninvasiveness and low price, the diagnostic value of ultrasound in the diagnosis of disease is increasing (23).

Pathologically, SPNs are similar to pancreatic neuroendocrine tumors, but it is sometimes difficult to distinguish them by routine pathology, and most of them can be distinguished by immunohistochemistry. The tumor AAT β-the expression rates of catenin, vimentin, CD56, and CD10 were high (15). With the deepening of research, it has been reported that P504S, TEF3, SOX-11, and progesterone receptors are also of great significance in identifying SPN (24). Immunohistochemical findings in this group of cases showed that β-catenin, vimentin, CD56, and CD10 were all positive, ae1/ae3 were positive in 12 cases (66.67%), and CgA were all negative.

At present, surgical resection of tumors is the only effective way to treat the disease. At present adjuvant therapy is reported in individual cases, and the drugs used are not the same. All kinds of guidelines actively recommend surgical treatment, but the scope and method of surgical resection are not standardized at present. The postoperative life expectancy for children is longer than that of adults, so more destructive surgical procedures, such as standard pancreaticoduodenectomy and combined splenectomy, should be used with caution. The biological characteristics of SPNs are potentially malignant or low-grade malignancy, poor invasiveness, and low-metastasis and low-recurrence rates (25). Even if there is metastasis or recurrence after surgery, there is still a chance of long-term survival (26). Therefore, under the condition of full evaluation before the operation, we should try our best to choose the operation plan that can remove the tumor as completely as possible and preserve the function of the pancreas. Lymph node metastasis is rare in SPNs, so it is not necessary to routinely dissect the surrounding lymph nodes (6).

For SPNs located in the head of the pancreas, in addition to traditional pancreaticoduodenectomy, pancreaticoduodenectomy with preservation of the common bile duct, pancreaticoduodenectomy with preservation of the pylorus, and resection of the head of the pancreas with preservation of the duodenum can be selected (27). If the capsule is intact, simple enucleation of the tumor can be considered for exogenous tumors located at the head of the pancreas. Some studies suggest that duodenum-preserving pancreatectomy is feasible if the tumor is <3 mm from the main pancreatic duct and ≥3 mm from the common bile duct (28). Simple tumor enucleation can be used for an SPN located in the neck of the pancreas if it is exogenous and the capsule is intact. In contrast, middle pancreatectomy (central pancreatectomy, CP) is feasible and suitable for the location of the tumor in the neck or proximal body of the pancreas. Some studies have shown that compared with distal pancreatectomy, CP does not increase postoperative complications and to some extent reduces the risk of pancreatic exocrine dysfunction caused by surgery (29). For SPNs in the tail of the pancreas or body and tail of the pancreas, distal pancreatectomy with preservation of the spleen should be the first choice. If the tumor is exogenous and the capsule is intact, simple enucleation of the tumor should be the first choice.

The spleen is an important immune organ that may cause explosive infection and septicemia after splenectomy, so preserving the spleen is more important for children than adults. If the adhesion between the tumor and the splenic vein is serious, a combined splenectomy can be selected when it is difficult to separate during the operation. However, during the operation, we can also try to remove the splenic vein and preserve the splenic artery and spleen. The difficulty of this operation is to deal with the splenic vessels well. If the splenic vessels cannot be treated properly, it may lead to splenic infarction and splenic abscess (30). Because of the difficulty in separating splenic vessels from the diseased tissues, the guardians of four children with splenectomy chose the scheme of combined splenectomy after full communication with their guardians.

With the increasing maturity and popularity of laparoscopic technology, an increasing number of operations are being performed through laparoscopy (31). Compared with open surgery, laparoscopic surgery has the following advantages: (a) the operation has the advantages of less trauma, less postoperative pain, beautiful wounds, and quick recovery. (b) Laparoscopy can enlarge the field of vision of surgery, which is beneficial to fine operation. (c) It can reduce intestinal adhesion and abdominal infection caused by the operation (32). From another point of view, complex anastomosis and digestive tract reconstruction under laparoscopy have no advantage. The intraperitoneal space of children is smaller than that of adults, which makes complex operations more limited. Although in theory, all kinds of SPN operations can be completed under laparoscopy, the lesion is located in the distal part of the pancreas or is more suitable for laparoscopic surgery.

## Conclusion

Solid pseudopapillary neoplasm is a rare and potentially malignant tumor in children. Its clinical manifestations are not specific, and there is no specific laboratory examination. Preoperative diagnosis depends mainly on imaging examination, and the gold standard of diagnosis is histopathological examination. The degree of malignancy of SPNs is low, and surgical resection can achieve a clinical cure. Therefore, to completely remove the tumor, retain glandular function as much as possible, and reduce postoperative complications, we should strictly grasp the surgical indications and formulate a reasonable surgical plan so that children can benefit more from the process of diagnosis and treatment.

## Data availability statement

The original contributions presented in the study are included in the article/supplementary material, further inquiries can be directed to the corresponding author.

## Ethics statement

The studies involving human participants were reviewed and approved by Medical Ethics Committee of the First Affiliated Hospital of Xinjiang Medical University (K2021110-05). Written informed consent to participate in this study was provided by the participants' legal guardian/next of kin. Written informed consent was obtained from the minor(s)' legal guardian/next of kin for the publication of any potentially identifiable images or data included in this article.

## Author contributions

AM and HW: conducted the study, collected, analyzed and interpreted the data, and wrote the manuscript. YW: statistically analyzed and interpreted the data. WL: planned the project and reviewed the manuscript. All authors contributed to the article and approved the submitted version.

## Conflict of interest

The authors declare that the research was conducted in the absence of any commercial or financial relationships that could be construed as a potential conflict of interest.

## Publisher's note

All claims expressed in this article are solely those of the authors and do not necessarily represent those of their affiliated organizations, or those of the publisher, the editors and the reviewers. Any product that may be evaluated in this article, or claim that may be made by its manufacturer, is not guaranteed or endorsed by the publisher.
